# Design, synthesis and *in vitro* and *in vivo* biological evaluation of flurbiprofen amides as new fatty acid amide hydrolase/cyclooxygenase-2 dual inhibitory potential analgesic agents

**DOI:** 10.1080/14756366.2021.1875459

**Published:** 2021-04-26

**Authors:** Alessandro Deplano, Jessica Karlsson, Federica Moraca, Mona Svensson, Claudia Cristiano, Carmine Marco Morgillo, Christopher J. Fowler, Roberto Russo, Bruno Catalanotti, Valentina Onnis

**Affiliations:** a Unit of Pharmaceutical, Pharmacological and Nutraceutical Sciences, Department of Life and Environmental Sciences, University of Cagliari, Monserrato, Italy; b Department of Integrative Medical Biology, Umeå University, Umeå, Sweden; c Department of Pharmacy, University of Naples Federico II, Naples, Italy; d Net4Science srl, University “Magna Graecia”, Catanzaro, Italy; e Drug Discovery Unit, Wellcome Centre for Anti-Infectives Research, School of Life Sciences, University of Dundee, Dundee, UK

**Keywords:** Flurbiprofen amides, FAAH inhibition, fatty acid amide hydrolase, endocannabinoid, cyclooxygenase, non-steroidal anti-inflammatory drugs, hyperalgesia, allodynia

## Abstract

Compounds combining dual inhibitory action against FAAH and cyclooxygenase (COX) may be potentially useful analgesics. Here, we describe a novel flurbiprofen analogue, N-(3-bromopyridin-2-yl)-2-(2-fluoro-(1,1'-biphenyl)-4-yl)propanamide (**Flu-AM4**). The compound is a competitive, reversible inhibitor of FAAH with a K_i_ value of 13 nM and which inhibits COX activity in a substrate-selective manner. Molecular modelling suggested that **Flu-AM4** optimally fits a hydrophobic pocket in the ACB region of FAAH, and binds to COX-2 similarly to flurbiprofen. *In vivo* studies indicated that at a dose of 10 mg/kg, **Flu-AM4** was active in models of prolonged (formalin) and neuropathic (chronic constriction injury) pain and reduced the spinal expression of iNOS, COX-2, and NFκB in the neuropathic model. Thus, the present study identifies **Flu-AM4** as a dual-action FAAH/substrate-selective COX inhibitor with anti-inflammatory and analgesic activity in animal pain models. These findings underscore the potential usefulness of such dual-action compounds.

## Graphical Abstract

**Figure:**
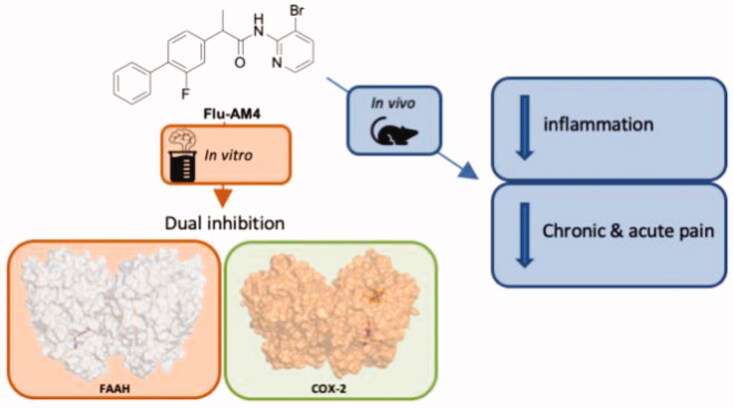

## Introduction

The endogenous cannabinoid system is involved in a number of physiological functions, including the regulation of pain^1^. Anandamide (AEA) and 2-arachidonoylglycerol (2-AG) are the two best-characterised endocannabinoids identified to date. They are synthesised on demand from membrane lipids, after which they are released and bind and activate the cannabinoid receptors (CB) that mediate multiple intracellular signal transduction pathways. Endocannabinoids are tightly regulated not only with respect to their biosynthesis but also with respect to their degradation. The two major hydrolytic enzymes responsible for the catabolism of AEA and 2-AG in the brain are fatty acid amide hydrolase (FAAH)^2^, and monoacylglycerol lipase (MAGL)^3^, respectively.

FAAH is a dimeric enzyme with a complex catalytic site characterised by three main interaction sites: the membrane access channel (MAC) where two charged residues favour the entrance of the polar head groups of fatty acid substrates, an adjacent “acyl-chain binding” cavity which likely contributes to the accommodation of substrates during catalysis, and a solvent-exposed “cytosolic port” (CP) which represents access to the cytosolic compartment of the cell. Once the substrate is bound to FAAH, the catalytic reaction occurs in the core of its binding site where an unusual catalytic triad (Ser241 − Ser217 − Lys142) performs the hydrolysis of the AEA. This occurs in two steps: firstly, a deprotonated Lys142 activates Ser241 through a proton exchange chain involving Ser217 leading to the protonation of Lys142. Then, the activated Ser241 attacks the substrate carbonyl group allowing the protonation of the leaving group ethanolamine which exits the enzyme through the CP^4^. Finally, a reverse proton transfer between Lys142 and Ser217 occurs, followed by a third proton transfer that re-establishes the original protonation state of the FAAH catalytic triad and leads to the formation of arachidonic acid.

Given the role of FAAH in the AEA hydrolysis, several classes of FAAH selective inhibitors, have been described in the scientific and patent literature^5^. FAAH inhibitors show efficacy in preclinical models of pain^6^^,^^7^, nausea^8^, and anxiety^9^^,^^10^ without the occurrence of tolerance^11^^,^^12^. However, FAAH inhibitors did not exhibit significant efficacy in several phase II clinical trials of osteoarthritis^13^, painful diabetic neuropathy^14^, pelvic pain and bladder dysfunction^15^ despite being well tolerated with little to no adverse events reported in each of these studies. One compound that was not well tolerated in man, however, was the irreversible FAAH inhibitor BIA10-2474, which left one person dead and four others with severe neurological symptoms^16^. However, these have been related to off-target effects due to the compound binds to a number of other serine hydrolases not exhibited by other FAAH inhibitors evaluated clinically^13^ and interactions with proteins other than FAAH are likely to mediate these extreme adverse events^17^. These clinical failures highlight the lack of translation between animal models and humans at least regarding the treatment of pain associated with inflammation. The real causes of the failure are not yet been identified, but it is reasonable that in a complex signalling pathway such as the endocannabinoid system, other metabolic enzymes and/or endocannabinoid targets may negate the effects of the FAAH inhibition.

The search for innovative strategies for pain management remains of key importance. In the literature, there are many examples of new potential analgesic molecules, which can selectively interact with known or new targets. Most of them are COX inhibitors^18^, TRPV antagonists^19^, or even FAAH inhibitors. Nevertheless, the usual approach of “one target-one drug” may not be the most optimal approach, which opens the way to the concept that molecules interacting with more targets that are possibly relevant for the same disease may show higher therapeutic effects and a safer biological profile. Therefore, in the last years, several studies have explored the dual targeting of FAAH and other related targets involved on signalling pathways of fatty acids and their derivatives, such as FAAH/MAGL inhibitors^20^, FAAH/TRPV1-receptor blockers^21^^,^^22^, FAAH/PGF_2α_-EA-receptor blockers^23^, and FAAH/COX-2 inhibitors^24–29^. In particular, the two latter strategies are based on the discovery that AEA and 2-AG are also COX-2 substrates that are converted to biologically active prostamides (prostaglandin ethanolamides) and prostaglandin glyceryl esters. Indeed, the prostamide F_2α_, produced by the cyclooxygenation of AEA, induces inflammatory and pro-algesic effects^30^. Therefore, dual blockade of FAAH and COX-2 could reduce prostamide biosynthesis while at the same time increasing AEA concentrations. In support of this hypothesis, it was seen in a key study that there was a synergistic interaction between the FAAH inhibitor URB597 and the non-steroidal anti-inflammatory drug (NSAID) diclofenac with respect to analgesia. Moreover, the co-administration also improved safety, whereby the FAAH inhibition lowered the incidence of ulceration produced by the NSAID^31^.

The development of dual FAAH/COX inhibitors started in 1997 when one of us found that a series of NSAID compounds inhibited anandamide deamidation albeit with a modest potency^32^^,^^33^. These findings, in particular for ibuprofen and flurbiprofen (**1**), led to the development of amide derivatives with increased FAAH potency while retaining COX-inhibitory potency. Among these, compounds deriving from the condensation of ibuprofen and flurbiprofen with 2-amino-3-methylpyridine (**Ibu-AM5** and **Flu-AM1**) were identified as the most promising dual inhibitors. In particular, **Ibu-AM5** was 2-3 orders of magnitude more potent than ibuprofen against FAAH^24^^,^^34^^,^^35^ albeit a weaker COX-1 and COX-2 than ibuprofen. **Flu-AM1** was about 60-fold more potent against FAAH than flurbiprofen, and was a substrate selective inhibitor against COX-2 in that it inhibited the cyclooxygenation of 2-AG more potently than the cyclooxygenation of arachidonic acid (AA) by either form of COX^24^^,^^26^^,^^34^. Using molecular docking, molecular dynamics and evaluation of free energy using a QM/MM approach, we found that **Ibu-AM5** and **Flu-AM1** showed very similar binding modes in the same site located between acyl binding channel (ACB) and the MAC^35^.

Although these FAAH/COX compounds look very promising *in vitro*, the only *in vivo* data so far presented were with **Ibu-AM5**, which showed efficacy in a model of visceral pain without producing ulcerations^21^^,^^22^. An alternative approach, to combine the elements of flurbiprofen and the irreversible FAAH inhibitor URB597, led to a FAAH/COX dual inhibitor that was active in models of inflammation and also did not produce ulcerations^36^, suggesting that this profile is a class effect of FAAH/COX dual inhibitors. Finally, a hydrogen sulphide releasing analogue of ketoprofen with enhanced FAAH inhibitory properties compared to ketoprofen has recently been shown to produce analgesic effects without gastrointestinal damage^37^.

With respect to improving the potency of our compounds, we found that for **Ibu-AM5**, the replacement of the 2-amino-3-methylpyridine moiety with a 2-amino-3-halolpyridine caused an increased potency towards FAAH and a selective inhibition of COX-2 when 2-AG is used as substrate^38^^,^^39^. In particular the *N*-(3-bromopyridin-2-yl)-2-(4-isobutylphenyl)propanamide (**Ibu-AM68**) and 2-(6-chloro-9*H*-carbazol-2-yl)-*N*-(3-chloropyridin-2-yl)propanamide (**Carpro-AM6**) showed the best dual FAAH/COX-2 inhibition activity. In the present paper we report the design, synthesis, and the *in vitro* and *in vivo* evaluation of the pharmacological activity of a new series of halogenated flurbiprofen amide derivatives, retaining the COX inhibition profile of the leads, but with an improved FAAH inhibition profile.

## Materials and methods

### General methods

Anandamide [ethanolamine-1-^3^H] (specific activity 2.22 TBq mmol-1) was purchased from American Radiolabeled Chemicals, Inc (St.Louis, MO). Ovine COX-1 (cat. no. 60100), human recombinant COX-2 (cat. no. 60122), COX-2 polyclonal antibody (rabbit antimouse, cat #: 160106), arachidonic acid, 2-AG, AEA and URB597 (cyclohexylcarbamic acid 30-carbamoylbiphenyl-3-yl ester) were purchased from the Cayman Chemical Co. (Ann Arbour, MI, USA). Substrates were dissolved in ethanol or DMSO as appropriate. Polyclonal goat anti-rabbit immunoglobulin/HRP was obtained from Dako (Glostrup, Denmark). Protease inhibitor cocktail set III was obtained from Merck Millipore (Darmstadt, Germany). All commercially available solvents and reagents were used without further purification and were purchased from Sigma-Aldrich (Milan, Italy). ^1^H-NMR spectra were recorded on an Inova 500 spectrometer (Varian, Palo Alto, CA), ^13 ^C-NMR spectra were recorded on a Bruker Advance III HD 600. The chemical shifts (δ) are reported in part per million downfield from tetramethylsilane (TMS), which was used as internal standard, and the spectra were recorded in hexadeuteriodimethylsulphoxide (DMSO-d_6_). Infra-red spectra were recorded on a Vector 22 spectrometer (Bruker, Bremen, Germany). The main bands are given in cm^−1^. Positive-ion electrospray ionisation (ESI) mass spectra were recorded on a double-focusing MAT 95 instrument (Finnigan, Waltham, MA) with BE geometry. Melting points (mp) were determined on a SMP1 Melting Point apparatus (Stuart Scientific, Stone, UK) and are uncorrected. All products reported showed spectra in agreement with the assigned structures. The purity of the tested compounds was determined by combustion elemental analyses conducted by the Microanalytical Laboratory, Department of Chemical and Pharmaceutical Sciences of the University of Ferrara with a MT-5 CHN recorder elemental analyser (Yanagimoto, Kyoto, Japan) and the values found were within 0.4% of theoretical values.

### General procedure for synthesis of flurbiprofen amides

The solution of Flurbiprofen (**1**) (0.24 g, 1 mmol), EDC (0.19 g, 1.1 mmol) and HOBt (0.13 g, 1 mmol) in anhydrous MeCN (10 ml) was stirred at r.t., after 30 min the opportune amine (1 mmol). The mixture was stirred at r.t. for 72 h and the solvent was then removed under vacuum. The residue was dissolved in AcOEt (20 ml) and washed sequentially with brine (2 × 5 ml), 10% citric acid (2 × 5 ml), saturated NaHCO_3_ aqueous solution (2 × 5 ml) and water (2 × 5 ml). The organic layer was dried over anhydrous Na_2_SO_4_ and evaporated under vacuum to give the title amides.

#### 2–(2-Fluoro-(1,1'-biphenyl)-4-yl)-N-(3-(trifluoromethyl)pyridin-2-yl)propanamide (Flu-AM3)

Obtained following the general procedure by the condensation between flurbiprofen and 2-amino-3-(trifluoromethyl)pyridine. Yield 45%. m.p. 110–112 °C. ^1^H NMR (DMSO-d_6_) *δ* 1.45 (d, *J* = 7.0 Hz, 3H, CH_3_), 3.99 (m, 1H, CH), 7.28–8.73 (m, 11H, Ar), 10.39 (s, 1H, NH). IR (Nujol) 3267, 1673, 1583, 1515 cm^−1^. *m/z* 389 (M + H)^+^. Elemental analysis: calculated for C_21_H_16_F_4_N_2_O (388.37)% C 64.95; H 4.15; N 7.21; found % C 65.00; H 4.14; N 7.17.

#### N-(3-Bromopyridin-2-yl)-2-(2-fluoro-(1,1'-biphenyl)-4-yl)propanamide (Flu-AM4)

Obtained following the general procedure by the condensation between flurbiprofen and 2-amino-3-bromopyridine. Yield 60%. m.p. 89–90 °C. ^1^H NMR (DMSO-d_6_) *δ* 1.45 (d, *J* = 7.0 Hz, 3H, CH_3_), 3.99 (q, *J* = 7.0 Hz, 1H, CH), 7.23-8.42 (m, 11H, Ar), 10.35 (s, 1H, NH). ^13 ^C NMR (DMSO-d_6_) *δ* 18.4, 44.7, 115.2, 115.3, 115.4, 117.6, 124.2 (2 C), 128.0 (2 C), 128.8, 128.9 (2 C), 142.2, 143.2, 143.3, 147.7, 158.3, 159.9, 175.2. IR (Nujol) 3265, 1667, 1511 cm^−1^. *m/z* 399, 401(M + H)^+^. Elemental analysis: calculated for C_20_H_16_BrFN_2_O (398.26)% C 60.17; H 4.04; N 7.02; found % C 60.25; H 4.06; N 6.99.

#### N-(3-Chloropyridin-2-yl)-2-(2-fluoro-(1,1'-biphenyl)-4-yl)propanamide (Flu-AM6)

Obtained following the general procedure by the condensation between flurbiprofen and 2-amino-3-chloropyridine. Yield 60%. m.p. 61–63 °C. ^1^H NMR (DMSO-d_6_) *δ* 1.48 (d, *J =* 7.0 Hz, 3H, CH_3_), 4.01 (q, *J* = 7.0 Hz, 1H, CH), 7.30–7.56 (m, 9H, Ar), 7.98 (d, *J* = 8.0 Hz, 1H, Ar), 8.40 (m, 1H, Ar), 10.41 (s, 1H, NH). ^13 ^C NMR (DMSO-d_6_) *δ* 18.4, 44.6, 115.1, 115.3, 123.3, 124.2 (2 C), 127.9 (2 C), 128.7, 128.8 (2 C), 130.6, 130.7, 138.9, 147.0, 148.0, 158.1, 159.8, 171.9. IR (Nujol) 3280, 1671, 1578 cm^−1^. m/z 327, 329 (M + H)^+^. Elemental analysis: calculated for C_20_H_16_ClFN_2_O (326.75)% C 67.70; H 4.55; N 7.90; found % C 67.63; H 4.54; N 7.93.

### Computational methods

#### QM optimisation and electrostatic potential

Quantum mechanics (QM) calculations have been used to evaluate the strength of interaction between the ligands (**FluAM-1** and **FluAM-4**) and the rat FAAH (*r*FAAH) Phe381 sidechain, and to analyse the electrostatic potential surface (ESP) of the compounds. The starting conformation was retrieved from the published **FluAM-1** binding mode^35^. The Phe381 and fluoro-biphenyl moieties of the ligand were fixed during QM optimisation to adjust the geometry of the complex ligand:Phe381 using Gaussian software^40^ at B3LYP/6–311 + G(d,p) level of theory. Interaction energy (*Δ*E) between a ligand (L) and the Phe381 (P) was calculated as:

$$\Delta \text{E}={\text{E}}_{\text{LP}}–\left({\text{E}}_{\text{L}}+{\text{ E}}_{\text{P}}\right)$$

where E_LP_, E_L_ and E_P_ are the energies of the ligand:Phe381 complex, the ligand alone and the Phe381 alone, respectively.

Electrostatic potential maps were generated on the optimised conformations of the compounds. ESP surfaces were represented in pymol 2.3.2^41^ using 0.004 isodensity surface and consequently the surfaces were mapped by colour where the ESP varies from red (the most negative) to blue (the most positive).

#### FAAH/COXs receptors and ligands preparation

The crystal structure of *r*FAAH was downloaded from the Protein Data Bank (PDB ID: 3QK5^42^). For the COXs isoforms, the ovine COX-1 PDB model 3N8Z^43^ and the mouse COX-2 PDB model 3RR3^44^ were selected. The presence of the non-selective NSAID Flurbiprofen as co-crystallized ligand was used as main selection criterion of the two isoforms. However, the human COX-2 complexed with flurbiprofen was not found, thus the mouse COX-2 was chosen as isoform-2 target, given its 87% identity with human COX-2. Both monomers A and B of *r*FAAH and COXs were treated with the Protein Preparation Wizard tool implemented in Maestro ver. 11.12^45^, in order to add all the hydrogen atoms and assign the correct bond orders. Subsequently, the co-crystallized ligands (QK5 and Flurbiprofen) and water molecules were removed. Residue Lys142 of *r*FAAH was considered in its deprotonated form, according to the proposed catalytic mechanism^46^. The 3D structure of **Flu-AM3-6** amides described above were built using the Graphical User Graphical User Interface (GUI) of Maestro ver. 11.12. Their protonation state at pH 7.4 in water has been calculated using the Epik module. Finally, each compound was then subjected to a minimisation protocol as described in our previous work^38^.

#### Molecular docking

To investigate in detail the recognition of the Flurbiprofen amide halogenated derivatives **Flu-AM3-6** to *r*FAAH and both COXs isoforms, docking simulations were carried out with the Glide software package, using the Standard Precision (SP) algorithm of the GlideScore function and the OPLS 2005 force field^47^. The docking binding site has been defined by means of a grid box of 29 × 29 × 29 Å centred in the ligand-binding cavity of *r*FAAH, and of 15 × 15 × 15 Å centred to cover the cyclooxygenase site of both COX-1 and COX-2. In addition, given the presence of halogen atoms in the designed ligands that could act both as halogen bond donors (between the σ-hole and a Lewis-base acceptor in the receptor) and hydrogen bond acceptors (between the lone pairs and an H-atom donor in the receptor), three different Glide protocols were adopted in the grid generation: *i)* default Glide parameters; *ii)* halogens treated as halogen bond donors; *ii)* halogens treated as hydrogen bond acceptors. A total amount of 200 ligand poses was generated and the conformational sampling of the ligand was enhanced by two times, as reported by the default setting of Glide. Docking conformations of **Flu-AM3-6** were then clusterized based on their RMSD cut-off of 2 Å.

#### Molecular dynamics (MD)

The poses 1 and 2 of the most interesting compound **Flu-AM4** retrieved by the docking procedure were submitted to 500 ns of MD simulation. The same docking pose of **Flu-AM4** obtained in monomer A was loaded also in monomer B, then each monomer has been treated separately for the MD analysis. MD was performed with the AMBER16 software^48^. Each complex was immersed in a pre-equilibrated octahedral box of TIP3P water molecules, and then the system was neutralised. The final systems contained about 106,000 atoms. All simulations were performed with the Amber *ff14SB* force field^49^ for the protein, while ligand charges were obtained by computing the ESP by means of the Gaussian09 package^40^ using a B3LYP/6–31G* DFT level of theory, and then the restrained electrostatic potential (RESP) charges^50^ were obtained by a two-stage fitting procedure using Antechamber^51^ and the General Amber Force Field (GAFF) force field^52^. The geometry of the two systems was minimised using convergence criterion for the energy gradient set to 0.01 kcal (mol.Å)^−1^ in three steps, which include: (*i)* minimisation of hydrogen atoms in the system (5000 steps of steepest descent and 10000 steps of conjugate gradient); (*ii)* minimisation of hydrogen atoms, water molecules and counterions (2000 steps of steepest descent and 18,000 steps of conjugate gradient); finally (*iii)* the whole system (2000 steps of steepest descent and 18,000 steps of conjugate gradient). Thermal and pressure equilibrations of the systems were performed in three steps: 200 ps increasing the temperature from 0 to 298 K restraining the protein backbone and ligand; then, 800 ps in which the restraining force was gradually decreased and, finally, 200 ps were performed in order to equilibrate the system density at constant pressure (1 bar), constant temperature (298 K). Production runs of 500 ns were then performed under NPT conditions and a time step of 2fs. Trajectories and data were processed and analysed using the *cpptraj* module^53^ and the Visual Molecular Dynamics (VMD) graphics^54^. Combined clusterization analysis was carried out with the DBSCAN algorithm^55^. All figures were rendered using PyMOL (http://www.pymol.org).

### Pharmacology

#### FAAH assay

Ethical permission for the *in vitro animal* experiments was obtained from the Umeå Ethical Committee for Animal Research, Umeå, Sweden (n° A67-09). Brains (minus cerebella) from adult Wistar or Sprague-Dawley rats (killed by decapitation) or from male B6CBAF1/J mice (killed by cervical dislocation), stored at −80 °C, were thawed, weighed and homogenised in cold 20 mM HEPES, 1 mM MgCl_2_, pH 7.0 and thereafter centrifuged at 35000 x g for 20 min at 4 °C. Homogenates were washed (by centrifugation followed by resuspension in the buffer) twice and incubated at 37 °C for 15 min in order to hydrolyse all endogenous FAAH substrates. After a further centrifugation, pellets were resuspended in 50 mM Tris-HCl buffer, pH 7.4, containing 1 mM EDTA and 3 mM MgCl_2_, and frozen at −80 °C in aliquots until used for the assay. For the FAAH assay^56^ test compounds, homogenates (usually 0.5–0.8 µg protein per assay, diluted with 10 mM Tris-HCl, 1 mM EDTA pH 7.4) and 25 µL of [^3^H]AEA in 10 mM Tris- HCl, 1 mM EDTA, pH 7.4, containing 1% w/v fatty acid-free bovine serum albumin, final substrate concentration of 0.5 µM) were incubated for 10 min at 37 °C (final assay volume 200 µL). Reactions were stopped by placing the tubes on ice. Final assay concentrations of the solvents used for the compounds (ethanol or DMSO) were in the range 1–5%. Activated charcoal (80 µL + 320 µL 0.5 M HCl) was added and the samples were mixed and left at room temperature for about 30 min. Following centrifugation at 2000 g for 10 min, aliquots (200 µL) of the supernatants were analysed for tritium content by liquid scintillation spectroscopy with quench correction. Blank values were obtained by the use of buffer rather than homogenate. In general, FAAH assays upon three homogenates were undertaken using separate inhibitor dilution series (from a stock solution), with 6 concentrations of inhibitor in half-log concentrations (i.e. 1, 3, 10 µM etc) ranging from 0.3–100 µM. Data were expressed as % of vehicle control and analysed using the algorithm log(inhibitor) vs. response - variable slope (four parameters) built into the GraphPad Prism computer program for the Macintosh (GraphPad Software Inc., San Diego, CA). Two different curve fits were chosen: one where the top (uninhibited) value was set to 100 and the bottom (maximum inhibition) was set to 0, and one where the top was set to 100 and the bottom allowed to float. The best model (when the bottom value returned >0) was chosen by Akaike’s informative criteria. For the compounds here, the simpler model was always returned. Since the program uses log_10_ inhibitor concentrations, the IC_50_ values for the inhibitable fraction are derived from the corresponding -log_10_(IC_50_) (pI_50_) values. Hence the SE values are for the pI_50_ values rather than the IC_50_ values. In consequence, we report both pI_50_ and IC_50_ values to indicate the SE values. With this assay, the selective FAAH Inhibitor URB597 completely blocked the hydrolysis of 0.5 µM [^3^H]AEA by rat brain homogenates at a concentration of 100 nM and caused 98% inhibition at 10 nM following a preincubation of 60 min between inhibitor and substrate, indicating that the assay as used here is specific for FAAH^57^. For the kinetic experiments, the data were analysed using algorithms for competitive, mixed-type or non-competitive inhibition in the GraphPad Prism program, and the best fit determined using Akaike's Informative Criteria.

#### COX-1 and 2 inhibition experiments

The assay was performed according to Meade et al.^58^ with minor modifications^27^. An oxygen electrode chamber with integral stirring (Oxygraph System, Hansatech Instruments, King’s Lynn, U.K.) was calibrated daily to ambient temperature and air pressure. The assay buffer contained 100 mM Tris-HCl buffer pH 7.4, 1 µM haematin, 2 mM phenol, 5 mM EDTA, 10 µM substrate (arachidonic acid or 2-AG) (final assay volume was 2 ml). After addition of **Flu-AM4** dissolved in ethanol (final assay concentration 1%), a baseline was established for 5 min before initiation of reaction by addition of 200 units of either ovine COX-1 or human recombinant COX-2. The change in oxygen consumption as a measurement of enzyme activity was monitored for approximately 5 min.

### *In vivo* pharmacological activity

#### Animals

The behavioural experiments were performed on CD1 male mice (25–30 g Charles Rivers-Italy). They were housed in the animal care facility of the Department Experimental of Pharmacology, University of Naples. The animals were acclimated to their environment for 1 week and had *ad libitum* access to standard rodent chow pellets VRF1 (purchased from Special Diet Service-SDS). All behavioural tests were performed between 9:00 AM and 13:00 PM, and the animals were only used once. Procedures involving animals and their care were conducted in conformity with international and national law and policies (EU Directive 2010/63/EU for animal experiments). The procedures reported here were approved by the Institutional Committee on the Ethics of Animal Experiments (CSV) of the University of Naples “Federico II” and by the “Ministero della Salute”, protocol no. 2014–0084607. At the end of all experiments for all pain models, the animals were euthanized by CO_2_ overdose. As suggested by the animal welfare protocol, all efforts were made to minimise animal suffering and to use only the number of animals necessary to produce reliable scientific data.

#### Drug treatments

During this study, **Flu-AM4** was administered systemically (intraperitoneally) and locally (intraplantar 100 microg/paw). For systemic administration, the compound was dissolved in PEG400 20% v/v, TWEEN 80 5% (v/v) and sterile saline and used at different doses (1–3–10 mg/kg). Vehicle group was only treated with PEG, TWEEN and sterile saline. For intraplantar administration **Flu-AM4** was dissolved in PEG400 10% v/v, TWEEN 80 5% (v/v) and formalin 5% in sterile saline.

#### Formalin-evoked hind-paw licking

Mice received intraplantar injections of formalin in saline (10%,vol/vol; 10 µl/paw). Paw licking was monitored by an observer blind to the experimental treatment for periods of 0–15 min (early phase) and 15–60 min (late phase) immediately after formalin administration. **Flu-AM4** was administrated both by local injection (100 µg) and systemic administration (10 mg/kg).

#### Carrageenan-induced paw eodema

Paw edoema was induced by a subplantar injection of 0.1 ml of sterile saline containing 1% λ-carrageenan (Sigma Aldrich) into the right hind paw. Paw volumes were measured by a plethysmometer (Ugo Basile, Italy) at different times after the paw injection. The increase in paw volume was evaluated as the difference between the paw volume measured (Vm) at each time point (1, 2, 4, 6 and 48 h post carrageenan) and the basal paw volume (Vb) measured immediately before carrageenan injection (*Δ* volume = Vm – Vb).

#### Mechanical hyperalgesia

Paw withdrawal threshold (PWT) to mechanical pressure was measured with a Randall-Selitto Analgesy-Meter for mice (Ugo Basile, Italy). PWT was measured on the ispsilateral paw at 1, 2, 4, 6 and 48 h post carrageenan injection. Each paw was tested twice per session. Cut-off force was set at 100 g.

#### Chronic constriction injury (CCI) model of neuropathic pain

Neuropathic pain was induced by ligation of the sciatic nerve as described previously^59^. Briefly, mice (*n* = 6 each group) were first anaesthetised with xylazine (10 mg/kg i.p.) and ketamine (100 mg/kg i.p.), and the left thigh was shaved and scrubbed with betadine, then a small incision in the middle left thigh (2 cm in length) was performed to expose the sciatic nerve. The nerve was loosely ligated at two distinct sites (spaced at a 2-mm interval) around the entire diameter of the nerve using silk sutures (7–0). The surgical area was closed and finally scrubbed with betadine. At day 7 and 14 Mechanical Allodynia (von Frey test) and Mechanical hyperalgesia (Randall-Selitto test) were assessed. In sham-operated animals, the nerve was exposed but not ligated.

#### Mechanical allodynia

To assess for changes in sensation or in the development of mechanical allodynia, sensitivity to tactile stimulation was measured using the Dynamic Plantar Aesthesiometer (DPA, Ugo Basile, Italy). Ligated animals were placed in a chamber with a mesh metal floor covered by a plastic dome that enabled the animal to walk freely, but not to jump. The mechanical stimulus was then delivered in the mid-plantar skin of the hind paw. The cut-off was fixed at 5 g, while the increasing force rate (ramp duration) was settled at 20 s. The DPA automatically records the force at which the foot is withdrawn and the withdrawal latency. Each paw was tested twice per session. This test did not require any special pre-training, just an acclimation period to the environment and testing procedure. Testing was performed on the ispsilateral (ligated) paw at day 7 and 14 after ligation, 1 h post dose.

### Statistical analysis

In vivo data are presented as mean ± SEM. In CCI experiments, DPA data are presented as paw withdrawal threshold (g), cold allodynia data are presented as number of paw withdrawal. The significance of differences between groups was determined by one-way repeated measurements ANOVA followed by *post hoc* Bonferroni's test.

## Results and discussion

### Design

On the basis of our previous work on **Flu-AM1,** we have designed a novel series of **Flu-AM1** derivatives where the pyridine methyl is substituted by halogen atoms. The most stable binding mode of **Flu-AM1** obtained in the QM/MM calculation showed the methyl group of the methyl-pyridine moiety located into a hydrophobic pocket defined by the Met436, Leu404 and Phe381. Taking advantage of these finding, we computationally validated our design exploring the possibility of enhancing this interaction by substituting the methyl group with halogens, in order to exploit halogen bonding of the σ-hole of the halogen with the aromatic ring of Phe381. To evaluate this possibility, we transformed **Flu-AM1** into **Flu-AM4** and performed an DFT geometry optimisation with the program Gaussian at the B3LYP/6–311 + G(d,p) level of the conformation of the ligand. To this aim, we defined a simplified system containing only the ligand and Phe381 and performed a DFT optimisation to evaluate energetic differences in the CH-π interaction when transforming methyl to bromine. The results (Supporting information Figure S1 and Table S1) clearly showed the greater tendency of bromine-derivative than methyl derivative to interact with Phe381.

### Synthesis

**Scheme 1.**
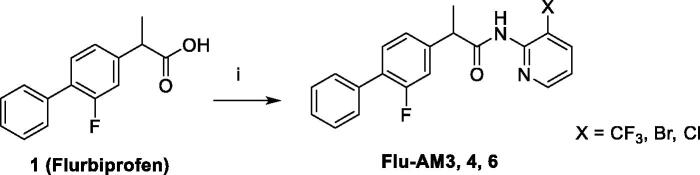
Synthesis of flurbiprofen amides: (i) EDCI, HOBt, MeCN, r.t. 72 h.

The target amides of flurbiprofen with halogenated pyridinamines were synthesised according to the Scheme 1. The amides **Flu-AM 3, 4, 6** were obtained by coupling the flurbiprofen (**1**) with 2-amino-3-halopyridines in the presence of 1–(3-dimethylaminopropyl)-3-ethylcarbodiimide hydrochloride (EDCI) and 1-hydroxybenzotriazole hydrate (HOBt) in dry acetonitrile (MeCN) solution. This synthetic pathway was found to be clean and high yielding.

### FAAH inhibition

**Figure 1.**
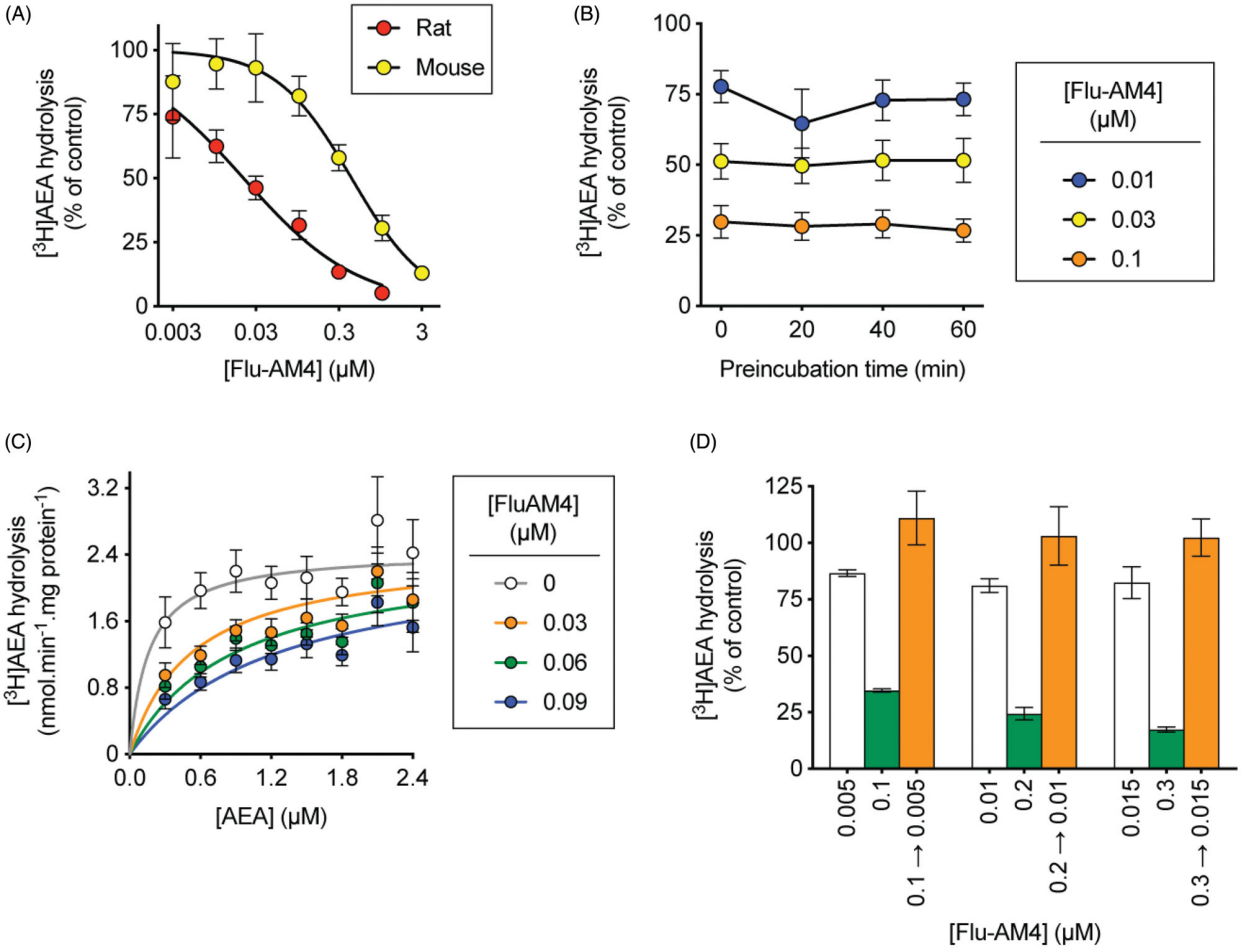
Mode of inhibition of FAAH by Flu-AM4 showing a reversible and competitive mode of FAAH inhibition. Inhibition of rat (unless otherwise shown) brain [^3^H]AEA hydrolysis by **Flu-AM4**. Panel A shows the inhibition curves for both rat and mouse brain homogenates from which the IC_50_ values were derived. In Panel B, rat brain homogenates were preincubated for the times shown prior to addition of substrate and incubation for a further 10 min. Panel C shows the kinetics of the inhibition of rat brain [^3^H]AEA hydrolysis by **Flu-AM4**. The data were better fitted by a model assuming competitive inhibition (K_i_ value 0.013 µM) than by a mixed model inhibition. All data are means ± s.e.m. (unless hidden by the symbols) *n* = 3–4. In Panel D, rat brain homogenates (at 20-fold normal strength) were preincubated with either vehicle, 0.1, 0.2 or 0.3 µM **Flu-AM4** for 60 min. Aliquots were then diluted 20-fold and assayed for FAAH activity. These are shown as 0.1 → 0.005, 0.2 → 0.01 and 0.3 → 0.015. Concomitantly, **Flu-AM4** was added to vehicle-preincubated aliquots to give concentrations of 0.005, 0.01 and 0.015 µM (representing free concentrations after a 20-fold dilution), 0.1, 0.2 and 0.3 µM final concentrations. The panel shows the data as % of corresponding control. For a fully reversible inhibitor, the preincubated + diluted samples should match the corresponding “diluted” rather than “concentrated” concentrations added after the preincubation phase (i.e. the 0.1 → 0.005 samples should match the 0.005 and not the 0.1 µM samples). Data are means ± s.e.m. (unless hidden within the symbols), *n* = 3–4.

The obtained halogenated amides (**Flu-AM3**, **4** and **6**), along with the reference compound **Flu-AM1** were evaluated for their ability to inhibit FAAH. The inhibition assays were performed using 0.5 µM [^3^H]AEA as substrate and rat or mouse brain homogenates as the enzyme source. The results of these primary assays are shown in Table 1 and Figure 1(A). The results revealed that the introduction of a halogenated moiety at 3-position of pyridine ring resulted in an increased ability to inhibit FAAH as compared to the reference **Flu-AM1**. All the new amides showed enhanced activity as compared with the reference. The replacement of the methyl on the pyridine ring of **Flu-AM1** with a trifluoromethyl group led to about 4-fold increase in activity (**Flu-AM3** IC_50_ 0.11 µM). The introduction in the same position of chlorine or bromine atom produced further increase in activity being amides **Flu-AM6** and **Flu-AM4** with nanomolar potency (IC_50_ 19 and 21 nM respectively, i.e. more than three orders of magnitude greater than flurbiprofen) and approximately 20-fold more potent than **Flu-AM1**.

**Table 1.** pI_50_ (-logIC_50_) and the derived IC_50_ values for inhibition of rat brain AEA hydrolysis by flurbiprofen amides. In all cases, the maximum inhibition was 100%.

Compound		pI_50_	IC_50_ (µM)
**Flurbiprofen**^a^		4.53 ± 0.02	29
**Flu-AM1**^a^		6.36 ± 0.02	0.44
**Flu-AM3**		6.96 ± 0.12	0.11
**Flu-AM4**		7.67 ± 0.10	0.021
**Flu-AM6**		7.72 ± 0.07	0.019

^a^The data for flurbiprofen and **Flu-AM1**, shown for comparative purposes, are from Ref. 26. Data are means ± s.e.m., *n* = 3–4.

### Mode of inhibition of FAAH by Flu-AM4

The mode of inhibition of FAAH by **Flu-AM4** is shown in Figure 1. The compound acted as a reversible inhibitor of rat brain FAAH (no time-dependent inhibition, loss of inhibition following dilution of the enzyme-inhibitor complex prior to addition of substrate). With respect to kinetics of inhibition, the **Flu-AM4** inhibition data were better fitted by a model assuming competitive inhibition (K_i_ value 13 nM) than by a mixed-model inhibition. The compound was, however, an order of magnitude more potent towards rat brain FAAH than mouse brain FAAH (pI_50_ 6.37 ± 0.10, IC_50_ value 430 nM). This is consistent with our previous work with **Ibu-AM5** and **Flu-AM1**^35^ and is presumably related to species differences of the amino acid compositions in the key binding sites of the enzymes. Species differences have also been reported for other types of FAAH inhibitor^60^^,^^61^. Nonetheless, **Flu-AM1** has sub-micromolar potency in the mouse, which is of relevance for *in vivo* studies.

### COX inhibitory activity of Flu-AM4

**Figure 2.**
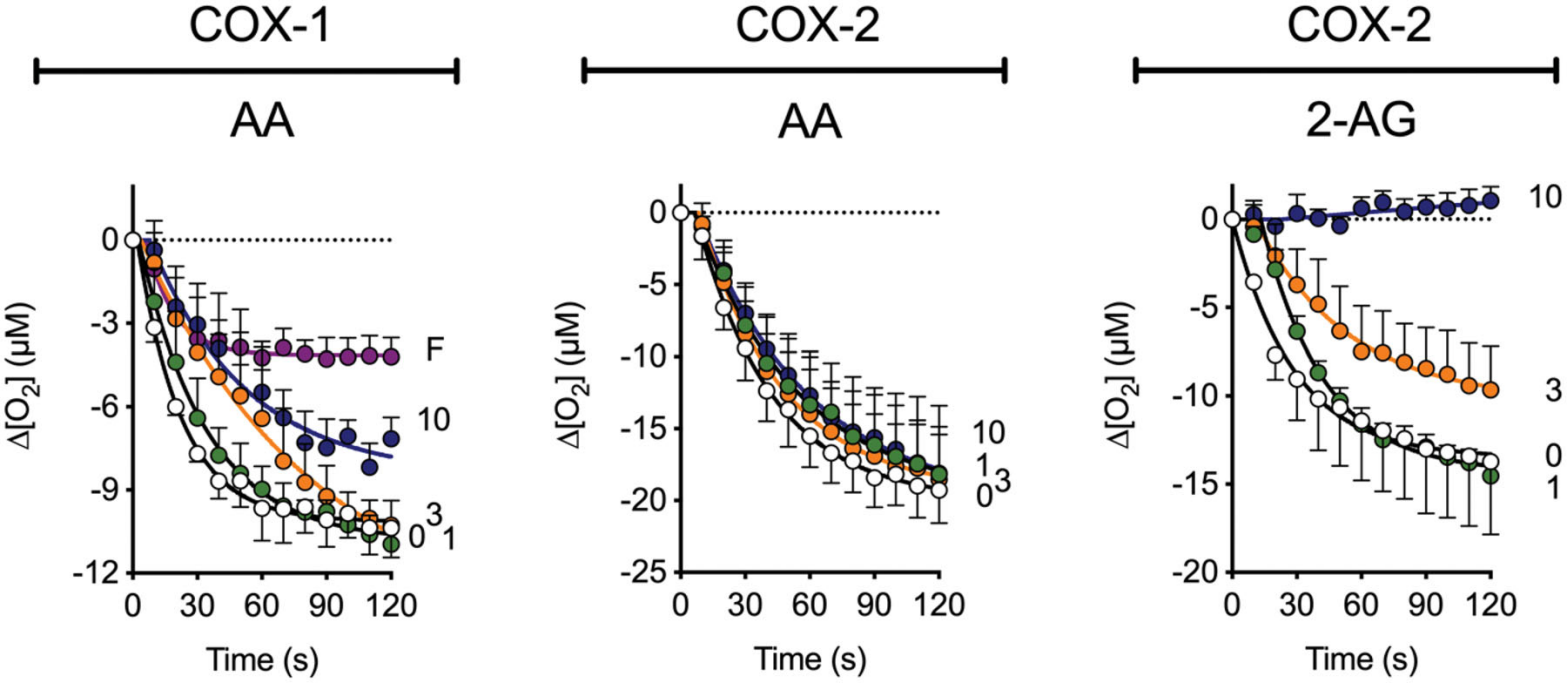
Inhibition of COX-1 and COX-2 by **Flu-AM4**. Inhibition of the activities of ovine COX-1 (towards arachidonic acid, AA) and human recombinant COX-2 (towards either arachidonic acid or 2-AG) by **Flu-AM4**. Shown are means ± s.e.m., *n* = 3 for the change in oxygen tension following addition of enzyme in the presence of the concentrations of the compounds (µM) shown on the right of each panel. The enzyme isoform and substrate (10 μM concentration) used are shown above each panel. “F” in Panel A refers to 10 µM flurbiprofen, used as a positive control for COX-1.

Given the ability of **Ibu-AM5** and **Flu-AM1** to inhibit COX^24^^,^^26^, we investigated the ability of **Flu-AM4** to inhibit COX-1 and COX-2 (Figure 2). Two substrates were investigated, arachidonic acid (AA, for both COX isoforms) and 2-AG (for COX-2; COX-1 does not catalyse the cyclooxygenation of this compound). At a concentration of 10 µM, **Flu-AM4** completely inhibited the cyclooxygenation of 2-AG by COX-2, while only partially inhibiting the cyclooxygenation of AA by COX-1 (2-AG is not a substrate of COX-1). This pattern of substrate selectivity is similar to that seen with flurbiprofen and **Flu-AM1**^23^.

On the basis of enzymatic assays, **Flu-AM4** was considered the most interesting derivative and investigated in vivo.

### Molecular modelling: FAAH inhibition

To study the effect of the substitution of the methyl group of **Flu-AM1** with halogens we performed a molecular docking investigation of the compounds **Flu-AM3-6** in monomer A of the crystal structure of rat FAAH (*r*FAAH) (PDB ID: 3QK5), with the Standard Precision (SP) algorithm of the Glide program (Schrödinger Inc.) by using three different protocols: *i)* default parameters *ii)* halogens treated as electron acceptors (halogen bond) and *iii)* halogens treated as hydrogen bond acceptors. The results of all the protocols, ranked together in both terms of Glide SP score and cluster population, clearly indicated as favoured poses those showing the biphenyl moiety pointing towards the catalytic triad and the pyridine ring entering the MAC (Supporting information Text S1.1, Table S2 and Figure S2), in a very similar orientation as found for **Flu-AM1** obtained after Molecular Dynamics (MD) refinement^35^.

Considering **Flu-AM4** as the most promising compound of the series, we refined the two best docking poses of **Flu-AM4** through 500 ns of classical MD simulations. The MD simulations were carried out on the *r*FAAH homodimer by loading the same docking pose of **Flu-AM4** obtained in both monomers A. MD analysis was performed considering the simulations of the two monomers as independent of each other, according to previous reports^62^. Therefore, four independent MD trajectories were analysed, two for each pose, for a total MD simulation time of 2 µs. The RMSD analysis of **Flu-AM4** revealed stable trajectories with small fluctuation within 1 Å of the ligand after an initial adjustment within the hydrophobic ACB channel (Supplementary Figure S3). The combined clusterization of the four trajectories, performed with the Density-Based Spatial Clustering of Applications with Noise (DBSCAN) algorithm^55^ showed that both docking poses converged to the same binding mode, represented by a main cluster accounting for 70% of the overall population (Table 2).

**Figure 3.**
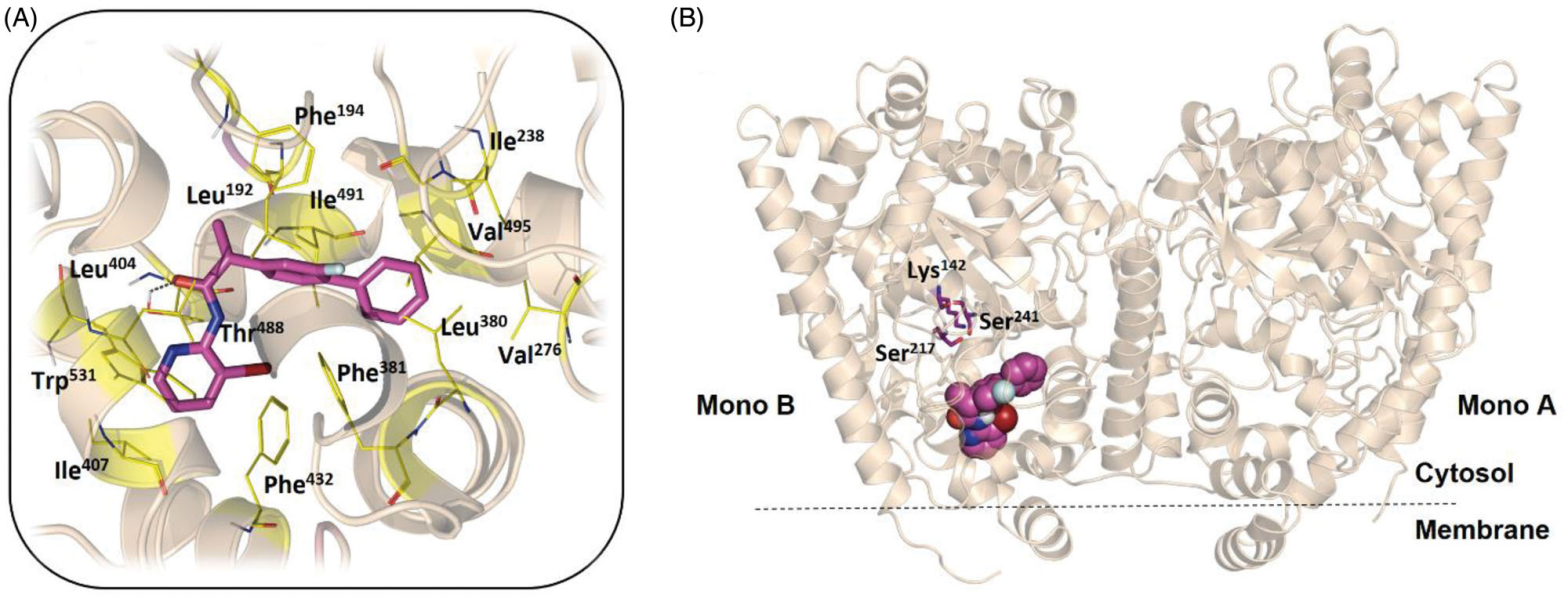
(A) The **Flu-AM4** L-shaped conformation (magenta stick) of the best cluster obtained from the DBSCAN analysis and minimised considering the explicit water molecules. (B) General view of the *r*FAAH enzyme (PDB ID: 3QK5) complexed with **Flu-AM4** in the same conformation (magenta spheres). *r*FAAH is displayed as wheat cartoon with the catalytic triad residues Lys142, Ser217 and Ser241 highlighted as magenta sticks. Amino acids involved in ligand/protein interactions with **Flu-AM4** are shown as yellow lines. Hydrogen bond interaction between the carbonyl of **Flu-AM4** and the hydroxyl group of Thr488 is shown as dashed black line.

The minimised representative structure of the best MD cluster (Figure 3(A)) showed an L-shaped conformation of the ligand and a binding mode mainly based on hydrophobic and aromatic interactions. The terminal phenyl ring fitted a hydrophobic pocket lined by Ile238, Val276, Ala377, Leu380, and Val495, with the central fluorine-substituted phenyl ring resulted stacked between two T-shaped π-π interactions, with Phe194 and Phe381, and flanked by Leu192 and Ile491. The bromo-pyridine ring showed hydrophobic contacts with Leu404, Leu429, Phe432, Leu433, Met436, Thr488 and a T-shaped π–π interaction with Trp531. Moreover, the bromine atom pointed towards Phe381, at a distance of 3.8 Å, thus indicating the possibility of a direct halogen bond interaction. Finally, a discontinuous hydrogen bond interaction (H-bond) was observed between the carbonyl of the ligand and the hydroxyl group of Thr488, with an occupancy percentage of 38% over the MD run. Taken together, the MD results suggested that the introduction of a bromine instead of the methyl on the pyridine ring induced a slightly different conformational behaviour, leading to a more compact structure that perfectly fits the hydrophobic environment of the ACB. The comparison of the proposed **Flu-AM4** binding mode to that previously published for **Flu-AM1** (Supporting information Figure S4) revealed how the different binding conformations of the two close compounds, likely driven by different internal interaction of the bromine or the methyl with the fluorine-substituted phenyl ring, determined a different ability to exploit the hydrophobic residue that characterises the ACB channel. In particular, the biphenyl moiety of **Flu-AM4** optimally fit a hydrophobic pocket establishing apolar contacts with 6 residues, while **Flu-AM1** only with 3 residues (Phe194, Ile238 and Ile491). Therefore, we can conclude that the introduction of the bromine enhanced the affinity for the FAAH, by means of the optimisation of hydrophobic interactions within the ACB channel.

**Table 2.** Combined cluster analysis of the MD simulation of pose 1 and pose 2 in monomers A and B.

Cluster	Population%	Population(%) Pose1_MonoA	Population(%) Pose1_MonoB	Population(%) Pose2_MonoA	Population(%) Pose2_MonoB
c0	70%	66%	19%	100%	95%
c1	16%	0%	64%	0%	0%
c2	12%	31%	15%	0%	0%

### Molecular modelling: COX-2 inhibition

**Figure 4.**
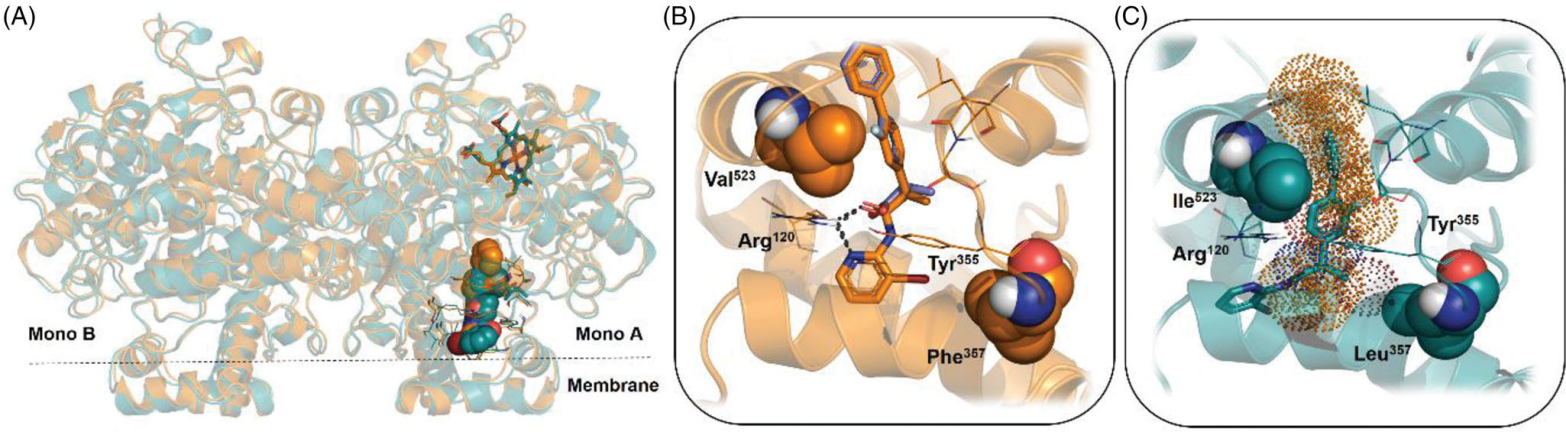
(A) General view of the COX-1 (dark-green cartoon) and COX-2 isoforms (orange cartoon) complexed, in the cyclooxygenase site, with the best docking results of **Flu-AM4** (dark-green and orange spheres, respectively in COX-1 and COX-2). (B) Best docking pose of **Flu-AM4** (orange stick) in the COX-2 isoform, superimposed with the co-crystallographic binding mode of flurbiprofen (violet stick) (PDB ID: 3RR3). (C) Best docking pose of **Flu-AM4** (dark-green stick) in the COX-1 isoform, superimposed with the binding mode of **Flu-AM4** against COX-2 (orange dots) to highlight the steric hindrance between the bromine atom and the Leu357, which hampers a full accommodation of **Flu-AM4** within the catalytic domain of COX-1. Key enzymes residues Arg120 and Tyr355 in COX-1 and COX-2 are shown as dark-green and orange lines, respectively. Amino acid residues influencing the binding mode of **Flu-AM4** between COX-1 and COX-2 are shown as spheres. Hydrogen bond interactions are shown as dashed black lines.

To study the molecular basis of COXs inhibition by **Flu-AM4**, docking experiments were carried out on COX-1 and COX-2 isoforms (PDB IDs: 3N8Z and 3RR3 respectively). Docking results of **Flu-AM4** in the COX-2 showed a clear preference for a single pose, strictly resembling the binding mode of the complexed flurbiprofen (**1**, Figure 4(B)), with the amide carbonyl oxygen and the pyridine nitrogen mimicking the interaction of the carboxylic acid group of **1**, directly engaging hydrogen bond interactions with the key residue Arg120. Accordingly, the analysis of the electrostatic potential surface of compounds **Flu-AM1** and **Flu-AM4** clearly showed a negative electrostatic potential localised between the pyridine nitrogen atom and the carbonyl group, supporting the hypothesis that the capability to retain COX-2 inhibitory properties is due to the peculiar behaviour of the amide-pyridine group to mimic the carboxylic group in NSAIDs. Interestingly, within the COX-1 **Flu-AM4** was not able to fit into the known active site occupied by **1**, lacking the key interactions with Arg120 (Figure 4(C)). In accordance with these observations, the resulting Glide SP scores are consistently higher in the case of COX-1 isoform (Supporting Information, Table S3). NSAIDs selectivity towards COX-2 has been achieved exploiting the different volume of the orthosteric binding site, mainly due to the presence of Ile523 in the catalytic domain of COX-1 (Val523 in COX-2), which reduces the volume of the cyclooxygenase site by closing the hydrophilic side pocket located above the Arg-120/Tyr-355 residues^63^. In our case, docking results of **Flu-AM4** against COX-1 highlighted also the role of Leu357 (Phe357 in COX-2) that further reduces the catalytic pocket, thus hampering the bromine-pyridine ring to adopt the same binding mode observed in COX-2 (Figure 4(C)).

### Flu-AM4 activity in acute and chronic pain mice models

The efficacy of FAAH inhibitors in several animal models of pain and inflammation have been described^6^^,^^63^^,^^64^. Most data are from irreversible inhibitors, but the reversible FAAH inhibitor OL-135 also reduces mechanical allodynia in a rat spinal nerve ligation model^65^. On the basis of enzymatic assays, **Flu-AM4** was considered the most interesting derivative and investigated *in vivo* in order to evaluate its anti-inflammatory and analgesic proprieties. In particular, we choose acute pain/inflammation animal models, such as carrageenan and formalin tests, to assess the ability of our compound to reduce oedema and inflammatory pain. Moreover, using the CCI model we have determinate the capability of **Flu-AM4** in a chronic neuropathic pain model.

**Figure 5.**
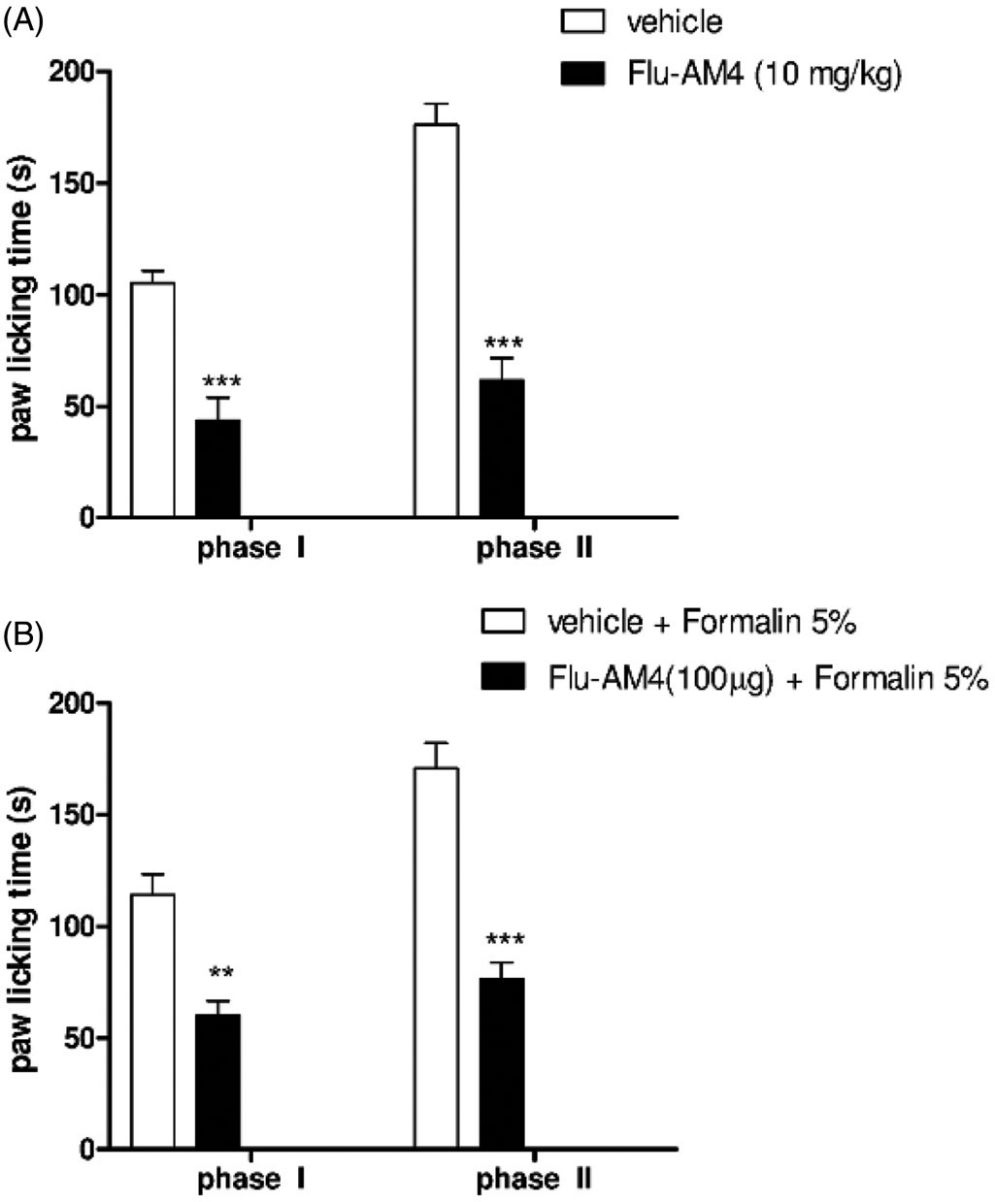
Effect of vehicle and **Flu-AM4** after A, systemic administration and B, local application in formalin-induced paw licking. In Panel A, 1 h before formalin injection, mice were treated with vehicle (white column) and **Flu-AM4** (10 mg/kg ip; black columns). In Panel B, mice were treated with vehicle + formalin in paw (white column) and **Flu-AM4** (100 µg) + formalin in paw (black columns). The cumulative licking time were counted for 15 min (phase I) and 50 min (phase II). Data are shown as mean ± SEM of 6 animals per group. ** *p* < 0.01 and *** *p* < 0.001 *vs.* vehicle, unpaired *t*-test.

The nocifensive response to formalin injection into the paw is characterised by two phases, an early sensorial phase (phase I) lasting the first 15 min, and a late inflammatory phase lasting from 20 to 60 min after the injection. This can be evaluated by determining the amount of time that the animals spend licking or biting the injected paw (Figure 5 white bars). Systemic **Flu-AM4** administration (10 mg/kg) was able to reduce the behavioural responses in both phases (Figure 5(A) black bars, ***p < 0.001). Moreover, we also evaluated the activity of **Flu-AM4** after local injection. Co-administration of formalin 5% and **Flu-AM4** (100 µg) reduced the paw licking time in both phases compared to formalin alone, suggesting that peripheral effects of the compound can contribute to its efficacy in this model (Figure 5(B) black bars, ***p < 0.001).

**Figure 6.**
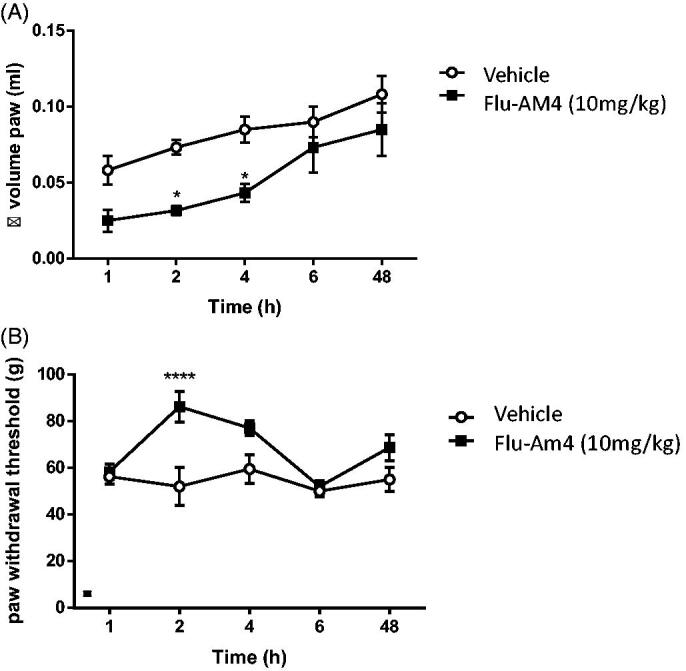
Effect of vehicle and **Flu-AM4** upon carrageenan-induced paw oedema (A) and paw hyperalgesia (B). Five min after carrageenan injection, mice were treated with vehicle (white circle) or **Flu-AM4** (10 mg/kg ip; black square). Data are shown as mean ± SEM of 6 animals per group. * *p* < 0.05, *** *p* < 0.001 *vs.* vehicle, unpaired *t*-test.

The anti-inflammatory properties of **Flu-AM4** were investigated further in the carrageenan paw oedema model. This is a classic inflammatory model in which injection of carrageenan induced plasma extravasation (oedema) and prolonged hyperalgesia until 72 h after injection. Systemic administration of **Flu-AM4** (10 mg/kg) reduced paw oedema at 2 and 4 h after carrageenan injection (Figure 6(A), *p < 0.05), moreover, the compound was able to reduce hyperalgesia seen 2 h after carrageenan (Figure 6(B), *p < 0.001).

**Figure 7.**
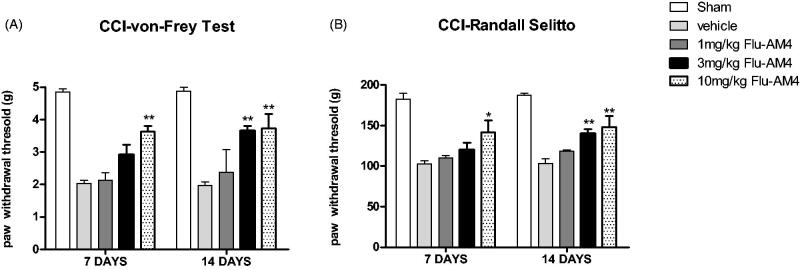
Effect of vehicle and **Flu-AM4** in CCI-induced mechanical (A) and hyperalgesia (B) allodynia on day 7 and 14. Mice 1 h before test were treated with vehicle (grey bars) and **Flu-AM4** (1, 3 or 10 mg/kg ip). Data are shown as mean ± SEM of 6 animals per group. *p < 0.05 and *p* < 0.01 vs. vehicle, 2-way repeated-measures ANOVA with *post-hoc* Bonferroni tests.

Finally, we evaluated **Flu-AM4** effect at different doses with respect to the mechanical allodynia and hyperalgesia produced in a neuropathic pain model, sciatic nerve ligation (CCI). We observed that 7- and 14-days vehicle CCI-treated mice (ctr) showed a significant reduction of the paw withdrawal threshold in both Von Frey and Randall-Selitto tests respect to sham not-ligated mice (Figure 7; *p* < 0.001 *vs.* sham). Systemic **Flu-AM4** treatment (1 h before test) significantly decreased the mechanical allodynia and hyperalgesia. In particular, the high dose, 10 mg/kg increased paw withdrawal latency in both test and in all experimental time (day 7 and 14), the dose of 3 mg/kg resulted efficacy only at 14 days in both tests and on both days 7 and 14. The dose of 3 mg/kg showed efficacy only at the 14-day time point in both tests, and the 1 mg/kg dose did not show any effect.

**Figure 8.**
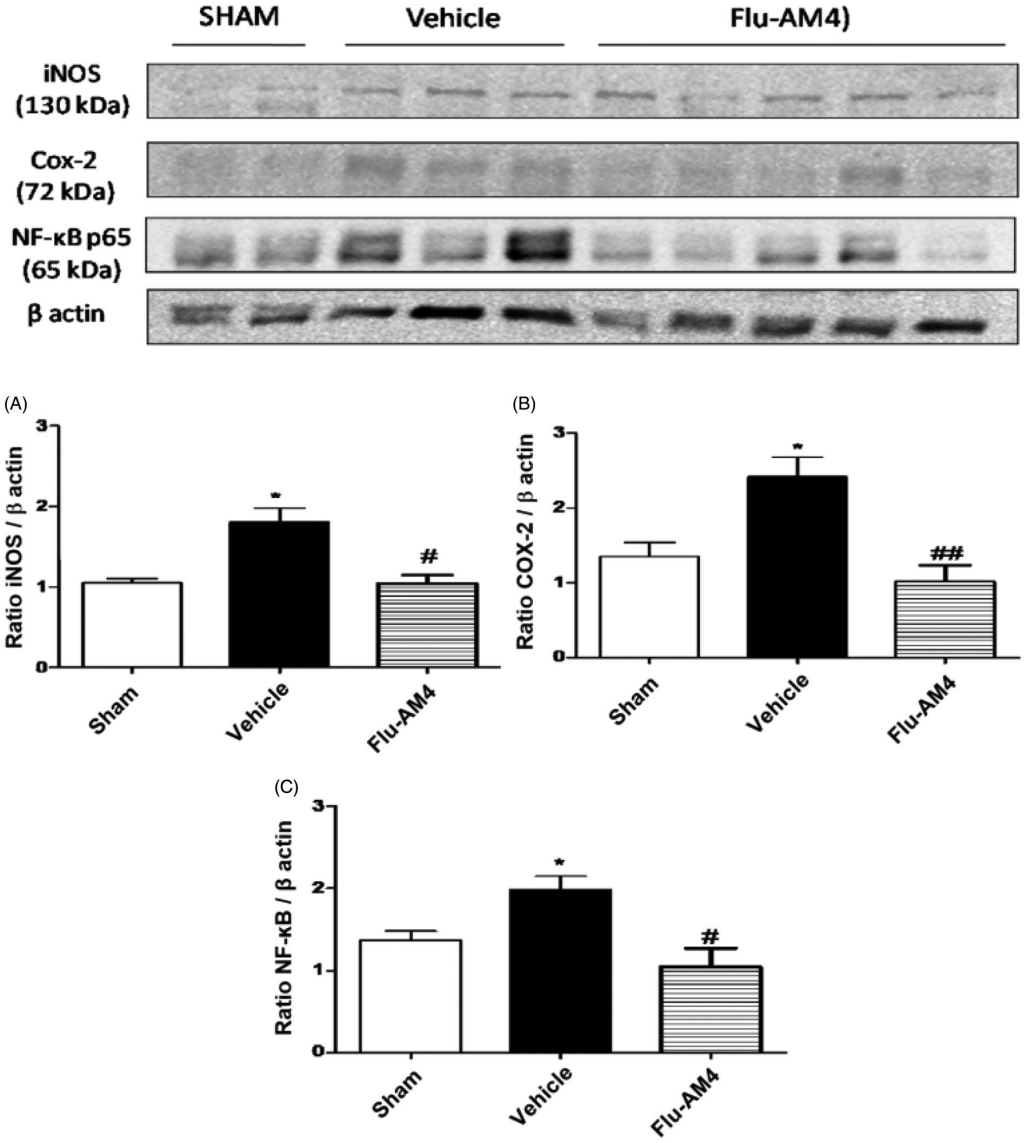
iNOS (A), COX-2 (B) and NF-κB-p65 (C) expression in the spinal cord of CCI-mice. Mice were treated 1 h before with vehicle (black bars) and **Flu-AM4** (10 mg/kg ip, grey bars). Sham mice represent animal without sciatic nerve ligation. Data are shown as mean ± SEM of 6 animals per group; representative blot are shown. *p < 0.05 vs. sham; #*p* < 0.05 and ##*p* < 0.01 *vs.* vehicle, one-way repeated-measures ANOVA with *post-hoc* Bonferroni tests.

We evaluated the expression of key factors involved in the development and maintenance of the inflammatory and pain state, such as COX-2, iNOS and NFκB in spinal cord of neuropathic-mice. Results clearly showed that sciatic nerve ligation induced an increase of iNOS (A), COX-2 (B) and NFκB-p65 (C) expression in the spinal cord. Acute **Flu-AM4** treatment significantly reduced this expression (Figure 8).

## Conclusion

A growing body of evidence suggesting that dual inhibition of COX and FAAH enzymes offers a potential therapeutic strategy to attenuate inflammatory and neuropathic pain states^24^^,^^31^^,^^36^. The development of dual FAAH-COX inhibitors would be of great potential value as a therapeutic strategy to treat different types of pain. Indeed, efforts are underway to synthesise such compounds^26^^,^^29^^,^^36^. In this paper we have described the design, the synthesis, the molecular mechanism of action and the pharmacological characterisation of a series of pyridine amide derivatives of flurbiprofen. In particular, enzymatic assays identified **Flu-AM4** the most promising compound within the series, docking and molecular dynamics studies suggested the molecular basis of dual action of pyridine-amide flurbiprofen derivatives. Moreover, our *in vivo* studies clearly indicated that the administration of a dual compound reduced inflammation and pain. **Flu-AM4**, indeed, was able to modulate the inflammatory process developed by carrageenan and formalin injection restoring the expression of important enzymes such as COX-2 and iNOS in the spinal cord of inflamed-mice. Moreover, **Flu-AM4** reduced also neuropathic pain by sciatic nerve ligation suggesting that its activity can influence much more elaborate and chronic pathways. Our results are in agreement with other studies where co-administration of the FAAH inhibitor URB597 and COX-1/2 inhibitor diclofenac produced synergistic antinociceptive effects in the acetic acid abdominal stretching assay^31^. Likewise, combination of the peripherally restricted FAAH inhibitor URB937 and the COX-1/2 inhibitor indomethacin significantly reduced nociceptive behaviour in the carrageenan and CCI models in a synergistic fashion^64^.

In conclusion, we have identified **Flu-AM4** as a dual-action reversible FAAH/substrate-selective COX inhibitor anti-inflammatory and analgesic activity in several animal pain models. These findings underscore the potential usefulness of such dual-action compounds and suggest that **Flu-AM4** is an ideal molecule with which to explore further this exciting possibility.

## Supplementary Material

Supplemental Material

## Supplementary data

These data include: docking studies on **Flu-AM3-6** in the rFAAH; QM/MM energetic and structural values, and molecular electrostatic potential surface calculated for the interaction of **Flu-AM1** and **Flu-AM4** with Phe381; comparison of **Flu-AM1** and **Flu-AM4** binding modes (PDF); Molecular model of **Flu-AM4** binding mode against FAAH (pdb); Molecular model of **Flu-AM4** binding mode against COX-2 (pdb).
