# Supplementary material for: Design, synthesis and in vitro and in vivo biological evaluation of flurbiprofen amides as new fatty acid amide hydrolase/cyclooxygenase-2 dual inhibitory potential analgesic agents
Source: J Enzyme Inhib Med Chem. 2021 Apr 26;36(1):940–53. doi: 10.1080/14756366.2021.1875459 (PMC8079065; doi:10.1080/14756366.2021.1875459)
Supplement: Supplemental Material [file IENZ_A_1875459_SM8909.pdf]

Appendix A. Supplemental material

Design, Synthesis and in Vitro and in Vivo Biological Evaluation of Flurbiprofen Amides as New Fatty Acid Amide Hydrolase / Cyclooxygenase-2 Dual Inhibitory Potential Analgesic Agents

Alessandro Deplano[a,§,], Jessica Karlsson[b,†,] Federica Moraca[c,d], Mona Svensson[b,†], Claudia Cristiano[c], Carmine Marco Morgillo[e], Christopher J. Fowler[b,†], Roberto Russo[c*], Bruno Catalanotti[c*] and Valentina Onnis[a]

[a]Department of Life and Environmental Sciences, Unit of Pharmaceutical, Pharmacological and Nutraceutical Sciences, University of Cagliari, University Campus, S.P. n° 8, Km 0.700, I-09042 Monserrato (CA), Italy
[b]Department of Integrative Medical Biology, Umeå University, Umeå, Sweden.
[c]Department of Pharmacy, University of Naples Federico II, Via D. Montesano 49, 80131 Naples, Italy
[d] Net4Science srl, University "Magna Græcia", Campus Salvatore Venuta, Viale Europa, 88100, Catanzaro, Italy
[e]Drug Discovery Unit, Wellcome Centre for Anti-Infectives Research, Division of Biological Chemistry and Drug Discovery, University of Dundee, DD1 5EH Dundee, United Kingdom

Contents

Page 2: **Text S1**. Docking studies on **Flu-AM3-6** in the *r*FAAH

Page 3: **Table S1**. QM/MM data calculated for the interaction ligand-Phe381.

Table S2. Docking results of **Flu-AM3**, **Flu-AM4** and **Flu-AM6**

Page 4: **Table S3.** Glide SP scores (kcal/mol) of **Flu-AM4** in the COX-2 and COX-1 isoforms.

Page 5: **Figure S1:** Molecular electrostatic potential surface (ESP) resulting from *ab-initio* calculations.

Page 6: **Figure S2**. Docking results of compounds **Flu-AM3-6**. (A-B-C).

Page 7: **Figure S3**. RMSD plots of **Flu-AM4** in both *r*FAAH monomers.

Figure S4: Superimposition between the **Flu-AM1** previously-published binding mode[1] and that of **Flu-AM4**.

Page 8: **References**

Text S1. Docking studies on Flu-AM3-6 in the *r*FAAH.

The three different docking protocols adopted for **Flu-AM4** were applied also to investigate the molecular recognition against the *r*FAAH of **Flu-AM3-6**. The results reported in Table S2 showed that all the three protocols gave similar results. In all cases we found, among the lowest score and most populated poses, two different orientations, similarly to **Flu-AM1** and **IbuAM5** [1]. In particular, docking poses were classified as *A-mode,* when the pyridine-amide moiety is pointing toward the catalytic triad (Lys142, Ser217 and Ser241) and the biphenyl/isobutyl moiety located close to the MAC channel, or *B-mode* when the biphenyl/isobutyl moiety is oriented toward the catalytic triad and the pyridine-amide moiety gets in the MAC channel. Globally, considering both the Glide SPscore and cluster populations, we observed that all the three Glide protocols clearly favored the *B-mode* (Table S2).

Analysis of the docking results of the three derivatives **FluAM-3-6** yielded two favored poses (Figure S2). The binding Pose 1 (Figure S2 A-B-C) resulted as the best Glide SP scored in case of **Flu-AM3** and **Flu-AM6**, and the second ranked in Glide SP score, but the most populated for **Flu-AM4**. Pose 1 resulted very similar (RMSD < 0.5 Å) among the three compounds with **Flu-AM3** differing only for the orientation of the fluorine atom in the biphenyl ring system. The binding Pose 1 showed the biphenyl moiety within the ACB channel with the second aromatic ring engaging a T-shape π-π interaction with Phe381 and hydrophobic contacts with Ile491, Leu192 and Leu380, while the halogen-substituted pyridine ring is sandwiched between the lipophilic pocket defined by Ile407, Leu429, Leu433 and Ile530. We also observed a hydrogen bond (H-bond) interaction of the carbonyl amide with Trp531. Binding Pose 2 (Figure S2 D-E-F) showed all the ligands in an orientation likely to the proposed binding mode for **Flu-AM1** [1], reporting little differences in the position of Flu-AM pyridine amide substituents. In particular, the electron-withdrawing group pointing toward the center of Fluorine-substituted phenyl ring in the case of **Flu-AM3** and **Flu-AM4** (Figure S2D and E, respectively), while the same is oriented in the opposite direction in case of **Flu-AM6** (Figure S2F). Furthermore, the region of the binding Pose 2 was more inside the MAC channel, with the biphenyl ring moiety lying in a hydrophobic pocket formed by Ile491, Ile238 and Phe244. The amide of the ligand is well stabilized by two H-bonds: the -NH of the ligand interacts with the main chain carbonyl of Thr488, and the amide carbonyl of the ligand interacts with the main chain -NH of Leu 401.

Table S1. QM/MM energetic and structural values calculated for the interaction ligand-Phe381. Interaction energies are calculated as the contribution of **Flu-AM1** and **Flu-AM4** binding

conformations with Phe381. Geometry properties are optimized distances and donor (Phe381 centroid (c):X-C, α_{don}) and acceptor (c-Phe381 edge Carbon (Ca):X, α_{acc}) angles. Energies are calculated at the B3LYP/6-311+G(d,p) level of theory.

	**ΔG (kcal/mol)**	**α_{don}(c:X-C)**	**α_{acc}(c-Ca:X)**	**Dist(X-c) (Å)**	**C-N-Ca-Na**
**Flu-AM1:Phe381**	-0.34	169.5	98.4	3.97	-44.7
**Flu-AM4:Phe381**	-1.06	163.4	96.1	3.78	-35.2

Table S2. Docking results of Flu-AM3, Flu-AM4 and Flu-AM6. Docking pose of Flu-AM4 submitted to MD refinements are highlighted in bold. The binding affinity computed by Glide SPscore is reported in kcal/mol.

Compound	Pose	Mode	Cluster populations	Glide SP score
***Flu-AM*3**	FLU-AM3R-c0-L	B	80	-9.69
	FLU-AM3R-c2-L-don	B	26	-9.15
	FLU-AM3R-c0-L-acc	B	30	-9.07
	FLU-AM3R-c1-don	B	30	-8.26
	FLU-AM3R-c6	B	10	-8.16
	FLU-AM3R-c0-don	A	33	-8.08
	FLU-AM3R-c0-acc	B	30	-7.85
	FLU-AM3R-c0-acc	B	30	-7.35
***Flu-AM*4**	****FLU-AM4R-L-c4-don**	**B**	**15**	**-9.25**
	***FLU-AM4R-c0**	**B**	**36**	**-9.21**
	FLU-AM4R-(2nd)-L	B	36	-9.15

	FLU-AM4R-c0-acc	B	42	-9.15
	FLU-AM4R-c0-don	A	40	-8.07
	FLU-AM4R-c5-don	B	13	-7.92
	FLU-AM4R-c3-acc1	B	20	-6.90
	FLU-AM4R-c3-acc2	B	20	-6.19
***Flu-AM*6**	FLU-AM6R-c0-L-don	B	35	-9.39
	FLU-AM6R-c0-L	B	46	-9.31
	FLU-AM6R-c0-L-acc	B	35	-9.24
	FLU-AM6R-c4	B	15	-7.91
	FLU-AM6R-c4-L	B	15	-7.81
	FLU-AM6R-c0-don	B	35	-7.74
	FLU-AM6R-c0-don	B	35	-6.88

*Binding Pose 1

**Binding Pose 2

Table S3. Glide SP scores (kcal/mol) of **Flu-AM4** in the COX-2 and COX-1 isoforms.

	Glide SP score	Glide Energy	Glide Emodel
**Flu-AM4 *vs* COX-2**	-9.41	-44.68	-68.52
**Flu-AM4 *vs* COX-1**	-4.91	-14.51	-19.39

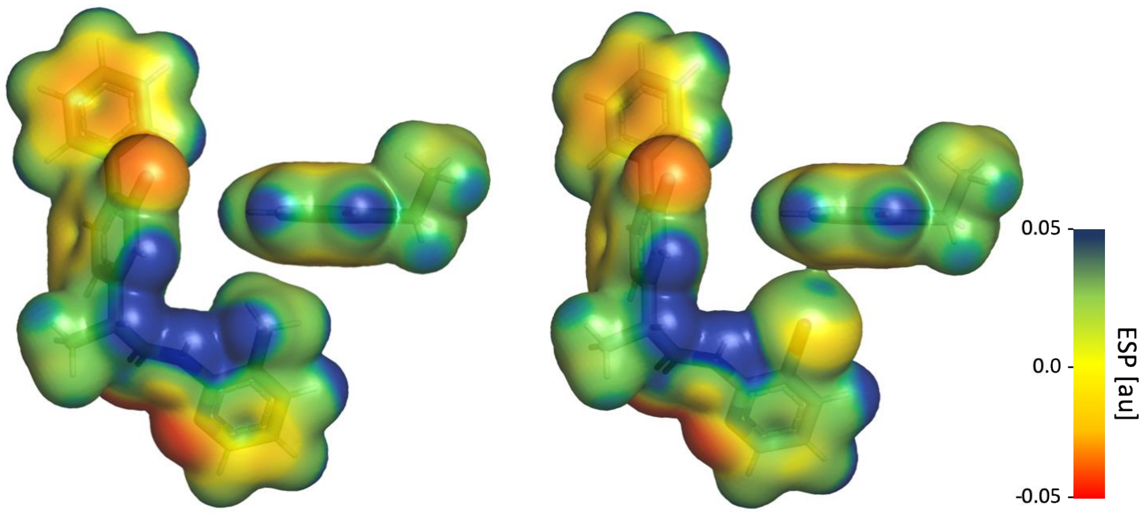

Figure S1: Molecular electrostatic potential surface (ESP) resulting from *ab-initio* calculations in atomic units on a surface of 0.004 au electron density (left, **Flu-AM1**:Phe381; right, **Flu-AM4**:Phe381). **Flu-AM1** starting conformation was taken from Ref. 1. **Flu-AM4** starting conformation was obtained by substituting the methyl group with a bromine atom in the **Flu-AM1** binding conformation**.**

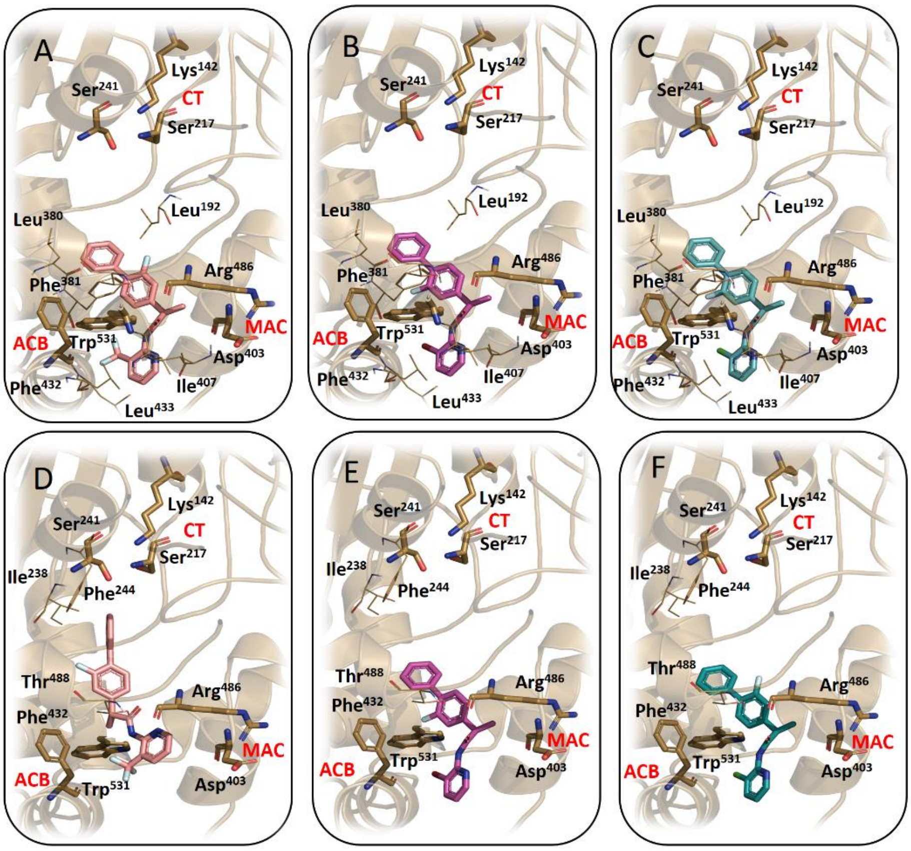

Figure S2. Docking results of compounds **Flu-AM3-6**. (A-B-C). Binding Pose 1 of **Flu-AM3** (pink stick); **Flu-AM4** (magenta stick) and **Flu-AM6** (cyan stick), respectively; (D-E-F) Binding Pose 2 of **Flu-AM3** (pink stick); **Flu-AM4** (magenta stick) and **Flu-AM6** (cyan stick), respectively. *r*FAAH receptor is displayed as wheat cartoon. Residues highlighting ACB, MAC and CT are represented in stick, while ligand-interacting aminoacids are shown as wheat lines.

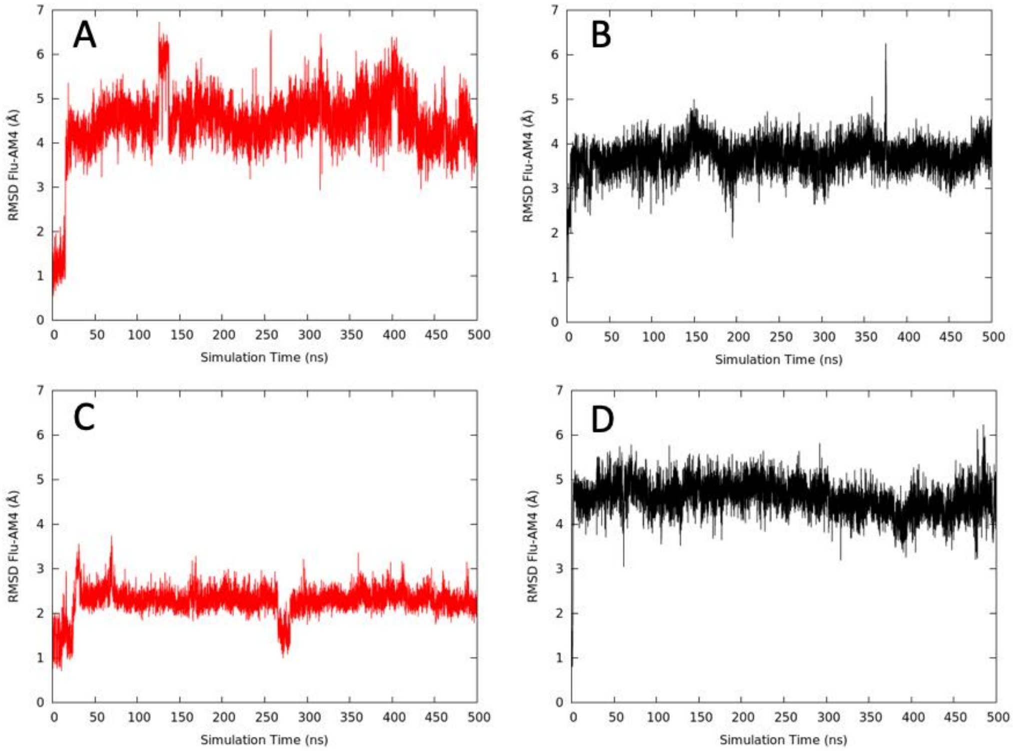

Figure S3. RMSD plots of **Flu-AM4** in both *r*FAAH monomers obtained during 500 ns of MD simulations: **A**) Binding Pose 1 in monomer A; **B**) Binding Pose 1 in monomer B; **C**) Binding Pose 2 monomer A; **D**) Binding Pose 2 monomer B. RMSD trends were obtained by superimposing each trajectory to the respective docking starting conformation.

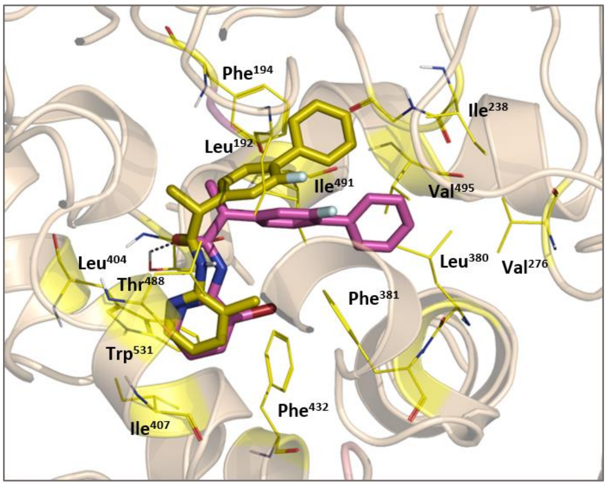

Figure S4: Superimposition between the **Flu-AM1** previously-published binding mode[1] and that of Flu-AM4 retrieved in this work. **Flu-AM1** and **Flu-AM4** are shown as dark-yellow and magenta stick, respectively.

References

1. Karlsson J, Morgillo CM, Deplano A, et al. Interaction of the n-(3-methylpyridin-2-yl) amide derivatives of flurbiprofen and ibuprofen with faah: Enantiomeric selectivity and binding mode. *PLoS One*. 2015;10(11):e0142711. doi:10.1371/journal.pone.0142711
